# A brief dataset on the model-based evaluation of the growth performance of Bacillus coagulans and l-lactic acid production in a lignin-supplemented medium

**DOI:** 10.1016/j.dib.2017.02.010

**Published:** 2017-02-11

**Authors:** Robert Glaser, Joachim Venus

**Affiliations:** Leibniz Institute for Agricultural Engineering and Bioeconomy (reg. assoc.), Max-Eyth-Allee 100, 14469 Potsdam, Germany

**Keywords:** L-Lactate, Glucose, Xylose, Arabinose, Lignin, Unstructured growth model, Cofermentation

## Abstract

The data presented in this article are related to the research article entitled “Model-based characterization of growth performance and l-lactic acid production with high optical purity by thermophilic *Bacillus coagulans* in a lignin-supplemented mixed substrate medium (R. Glaser and J. Venus, 2016) [1]”. This data survey provides the information on characterization of three *Bacillus coagulans* strains. Information on cofermentation of lignocellulose-related sugars in lignin-containing media is given. Basic characterization data are supported by optical-density high-throughput screening and parameter adjustment to logistic growth models. Lab scale fermentation procedures are examined by model adjustment of a Monod kinetics-based growth model. Lignin consumption is analyzed using the data on decolorization of a lignin-supplemented minimal medium.

**Specifications Table**

**Table:** 

Subject area	Biotechnology, Bioeconomy
More specific subject area	Cofermentation of hexoses and pentoses of lignocellulose hydrolyzates and lignin uptake by *Bacillus coagulans*.
Type of data	Table, Figure, Data file
How data was acquired	Conducting thermophile anaerobic fermentation of lignocellulose model substrate and lignocellulose hydrolysate.
	High pressure liquid chromatography for was used for carbohydrate analysis.
	UV–vis photometry was conducted for lignin analysis.
	Logistic and Monod models were used for the mathematical modeling and simulation approaches.
Data format	Raw, Filtered, Analysed
Experimental factors	Different *Bacillus coagulans* isolates were cultivated anaerobically in lignocellulose model substrate and enzymatic lignocellulose hydrolysate.
Experimental features	The growth behaviour and growth kinetics of different isolates of *Bacillus coagulans* were determined in micro-scale optical density measurements and lab-scale fermentations.
Data source location	Potsdam, Brandenburg, Germany, 52°26′17.9″N 13°00′48.2″E
Data accessibility	The data are available with this article.

**Value of the data**

• The data presents the kinetic analyses of three *Bacillus coagulans* strains co-fermenting lignocellulose-related sugars glucose, xylose, and arabinose.
• The presented data and methods can easily be used for benchmarking models for high-throughput optical-density screening procedures and growth performance of fermentation procedures.
• This data allows other researchers to compare fermentation results and model fitting directly and to extend the analyses.

## 1 Data

The dataset of this article provides information on the biotechnological production of lactic acid (LA) by different isolates of *Bacillus coagulans* grown on lignin-containing substrates. Screening data achieved by a high-throughput method to derive kinetic parameters for the evaluation of the resistance to the component alkali-lignin (AL) are given.

**Fig. 1:**
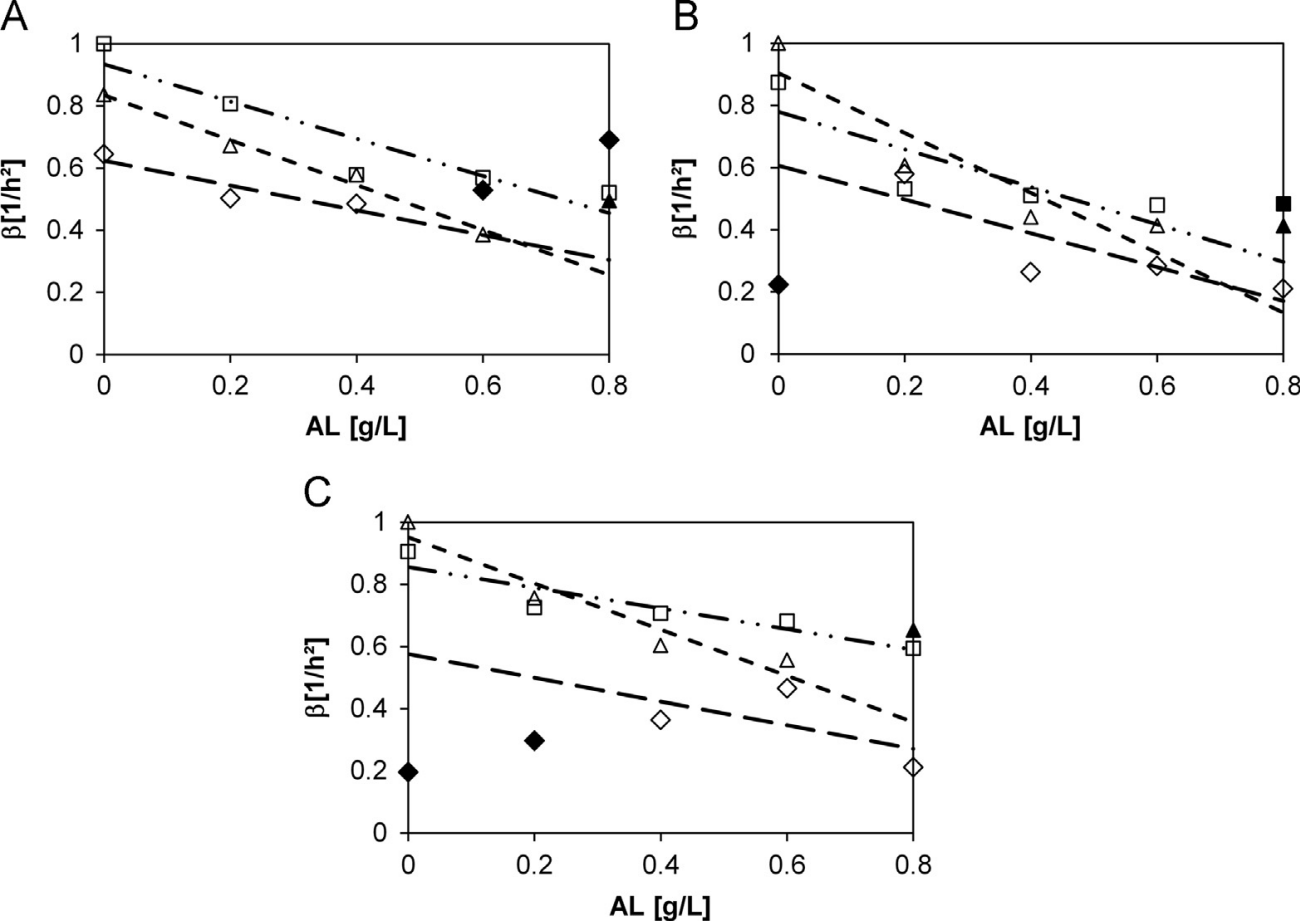
Growth prediction of *B. coagulans* strains based on the optical-density high-throughput screening expressed by the parameters *β* (□ glucose, ◊ xylose, Δ glucose+xylose). Linear regressions are indicated as lines (─ ▪ ▪ glucose, ─ ─ ─ xylose, − − − glucose+xylose). A: *β* of DSM No. 2314; B: *β* of DSM ID 14–298; C: *β* of DSM ID 14–301. Black symbols were not used for regression and extrapolation.

The data that are displayed in Fig. 1 represent the progression of parameter *β* [3] with increasing lignin concentration derived by the used screening method. The parameter *β* is discussed in the main research article Ref. [1] in comparison to a new parameter: *δ* which is also described in Ref. [1].

**Fig. 2:**
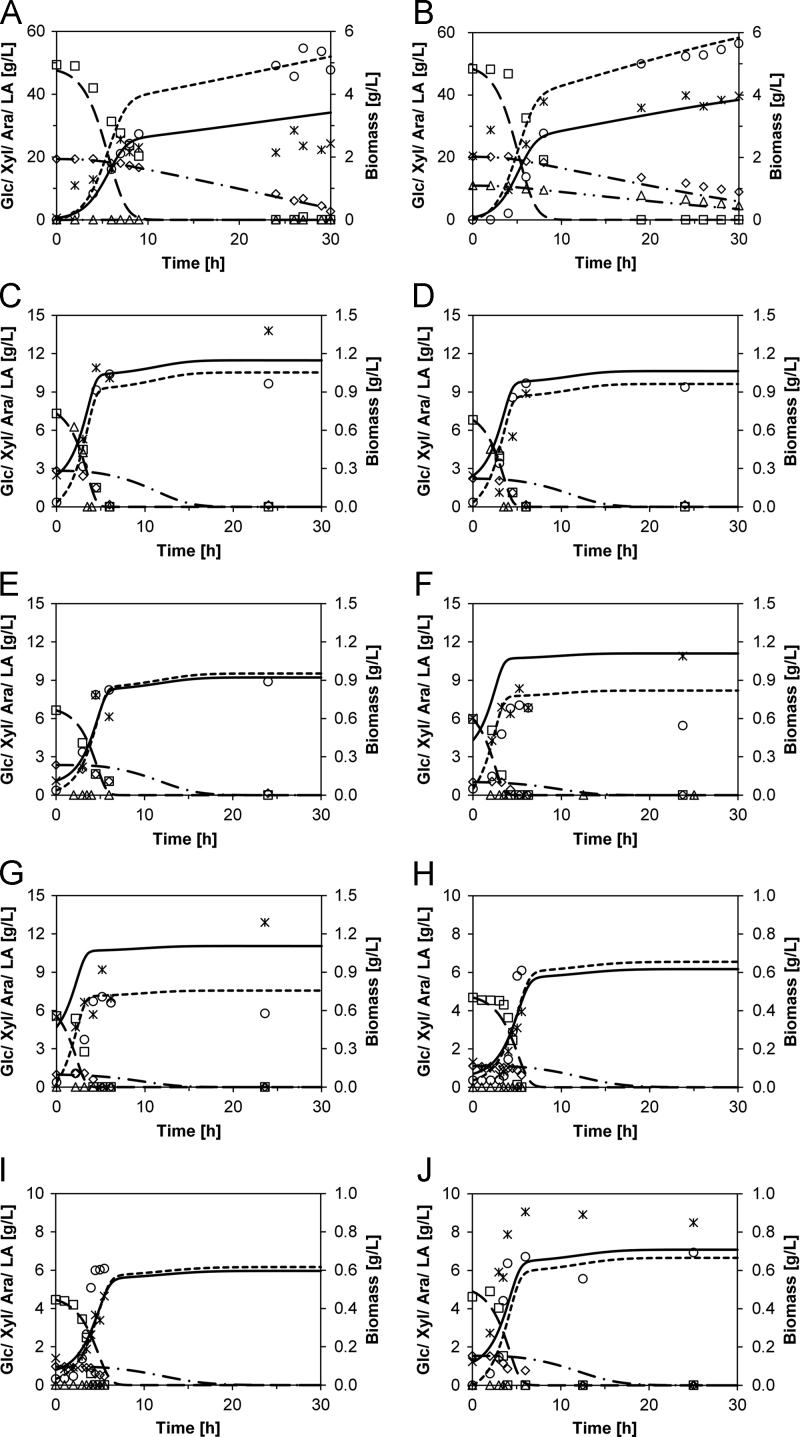
Fermentation time course data and mode prediction. A: DSM ID 14–298 grown in AM without AL and without arabinose and in B with arabinose. C, D, E: DSM No. 2314 grown in WSH, F, G grown in AWH, and H, I, J grown in PWH. Empirical results are displayed as symbols (□ glucose, ◊ xylose, Δ arabinose, ○ lactate, × biomass). Predictions are shown as lines (─ ─ ─ glucose, ─ ▪ ─ ▪ xylose, ─ ▪ ▪ arabinose, − − − lactate, ── biomass).

**Fig. 3:**
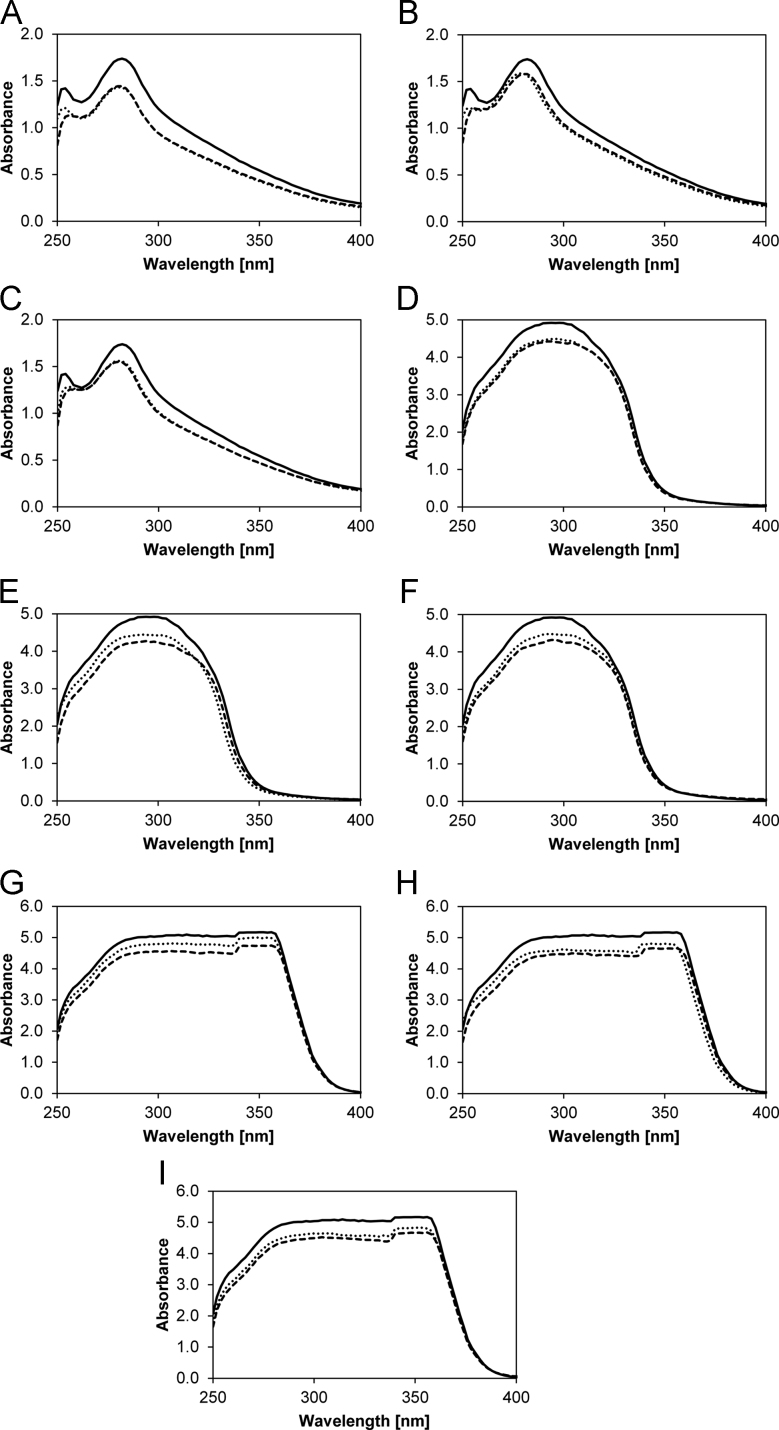
UV absorbance plots of the three selected lactic acid strains (A, D, G: DSM No. 2314; B, E, H: DSM ID 14–298; C, F, G: DSM ID 14–301) for the decolorization of A, B, C: AL solution, D, E, F: ferulic acid solution, and G, H, I: vanillin solution. (─ blank solution, ▪ ▪ ▪ 2.5 h sample, - - - 5 h sample).

**Fig. 4:**
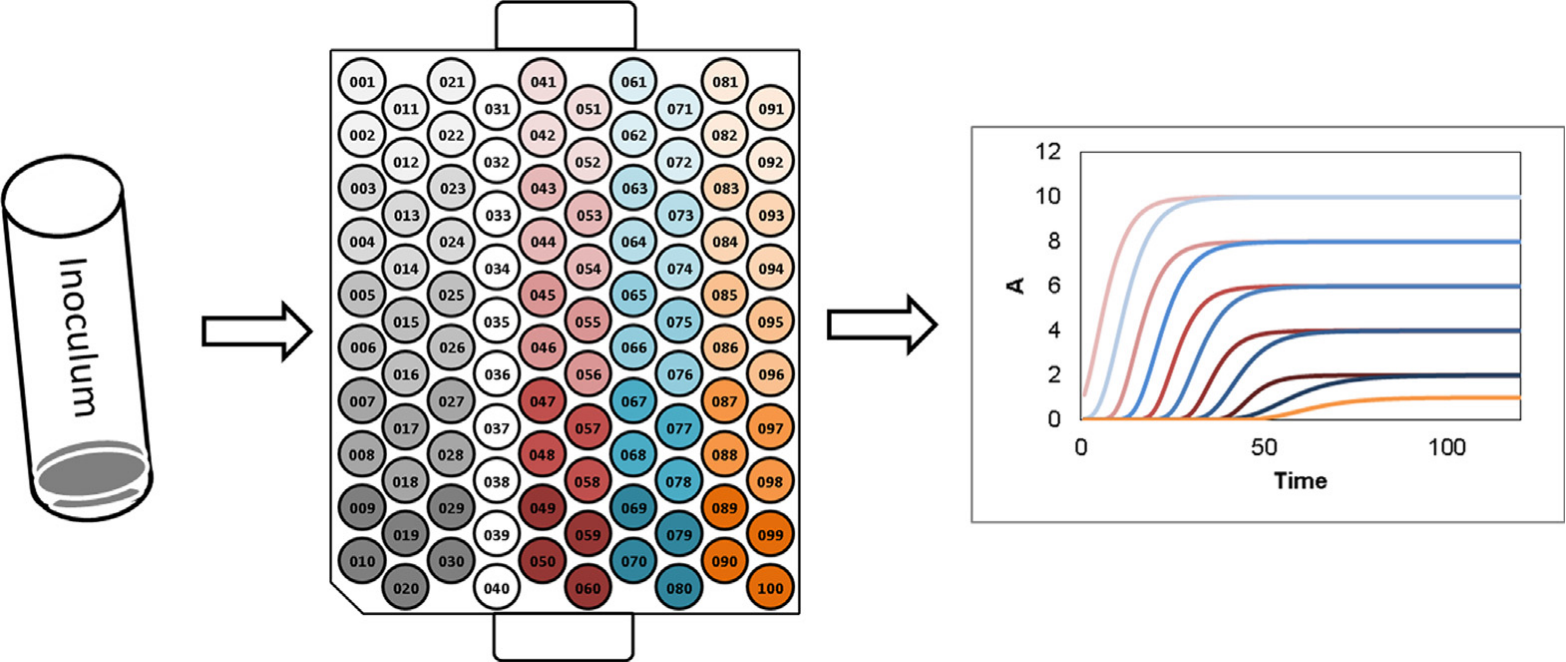
Experimental settings for the pipetting scheme of the Honeycomb plates.

The measurement data on a bacterial screening process, the derived parameter for the maximum growth rate *µ*_*max*_, and the lag time λ are given in the following files:

– Data shown in Fig. 1A and Fig. 1A in Ref. [1]:
  001 - Bioscreen turbidimetry measurements of lignin endurance - DSM No 2314.xlsx
– Data shown in Fig. 2A and Fig. 2A in Ref. [1]:
  002 - Bioscreen turbidimetry measurements of lignin endurance - DSM ID 14–298.xlsx
– Data shown in Fig. 3A and Fig. 3A in Ref. [1]:
  003 - Bioscreen turbidimetry measurements of lignin endurance - DSM ID 14–301.xlsx
  Basic data of lignocellulose hydrolysate fermentations using an artificial medium (AM), method is described in Ref. [1], and data of fermentations of wheat straw hydrolysate (WSH), aspen wood hydrolysate (AWH), and pine wood hydrolysate (PWH) is presented in Table 1 to give an overview of initial and stop conditions of fermentations.
  Additional fermentation data according Ref. [1] are given in the following files:
– Data shown in Figs. 2A and 6A in Ref. [1]:
  004 - Submerged fermentation - DSM No 2314 - 0.000 g per L Lignin.xlsx
– Data shown in Figs. 2B and 6A in Ref. [1]:
  005 - Submerged fermentation - DSM No 2314 - 0.625 g per L Lignin.xlsx
– Data not shown in Fig. 2 in Ref. [1]:
  006 - Submerged fermentation - DSM No 2314 - 1.250 g per L Lignin.xlsx
  007 - Submerged fermentation - DSM No 2314 - 2.500 g per L Lignin.xlsx
– Data shown in Figs. 3A and 6B in Ref. [1]:
  008 - Submerged fermentation - DSM ID 14–298 - 0.000 g per L Lignin.xlsx
– Data shown in Figs. 3B and 6B in Ref. [1]:
  009 - Submerged fermentation - DSM ID 14–298 - 0.625 g per L Lignin.xlsx
– Data shown in Figs. 3C and 6B in Ref. [1]:
  010 - Submerged fermentation - DSM ID 14–298 - 1.250 g per L Lignin.xlsx
– Data not shown in Figs. 3 and 6B in Ref. [1]:
  011 - Submerged fermentation - DSM ID 14–298 - 2.500 g per L Lignin.xlsx
– Data shown in Figs. 4A and 6C in Ref. [1]:
  012 - Submerged fermentation - DSM ID 14–301 - 0.000 g per L Lignin.xlsx
– Data shown in Figs. 4B and 6C in Ref. [1]:
  013 - Submerged fermentation - DSM ID 14–301 - 0.625 g per L Lignin.xlsx
– Data shown in Figs. 4C and 6C in Ref. [1]:
  014 - Submerged fermentation - DSM ID 14–301 - 1.250 g per L Lignin.xlsx
– Data shown in Figs. 4D and 6C in Ref. [1]:
  015 - Submerged fermentation - DSM ID 14–301 - 2.500 g per L Lignin.xlsx
– Data not shown in Fig. 4 in Ref. [1]:
  016 - Submerged fermentation - DSM ID 14–301 - 3.750 g per L Lignin.xlsx
  Derived parameters of the AM fermentation process were used to predict the behavior in actual lignocellulose hydrolysates of WSH, AWH, and PWH. The conducted fermentation procedures and predictions are shown in Fig. 2 and Table 2. The performance of the enzymatic hydrolyses are described in previous studies [3], [4].
  Additional fermentation data according Ref. [1] are given in the following files:
– Data shown in Fig. 2A:
  017 – Submerse fermentation - DSM ID 14–298 – Growth prediction in artificial medium without arabinose.xlsx
– Data shown in Fig. 2B:
  018 – Submerse fermentation - DSM ID 14–298 – Growth prediction in artificial medium.xlsx
– Data shown in Fig. 2C:
  019 - Submerse fermentation - DSM No 2314 - Wheat straw hydrolysate - 1.xlsx
– Data shown in Fig. 2D:
  020 - Submerse fermentation - DSM No 2314 - Wheat straw hydrolysate - 2.xlsx
– Data shown in Fig. 2E:
  021 - Submerse fermentation - DSM No 2314 - Wheat straw hydrolysate - 3.xlsx
– Data shown in Fig. 2F:
  022 - Submerse fermentation - DSM No 2314 - Aspen wood hydrolysate - 1.xlsx
– Data shown in Fig. 2G:
  023 - Submerse fermentation - DSM No 2314 - Aspen wood hydrolysate - 2.xlsx
– Data shown in Fig. 2H:
  024 - Submerse fermentation - DSM No 2314 - Pine wood hydrolysate - 1.xlsx
– Data shown in Fig. 2I:
  025 - Submerse fermentation - DSM No 2314 - Pine wood hydrolysate - 2.xlsx
– Data shown in Fig. 2J:
  026 - Submerse fermentation - DSM No 2314 - Pine wood hydrolysate - 3.xlsx
  Decolorization experiments with AL, ferulic acid (FA), and vanillin (VAN) were conducted. The data are shown in Fig. 3 and discussed in the main article Ref [1].
  The quantitative data on bacterial growth and the derived parameters are provided in the following files:
– Data shown in Fig. 3A:
  027 - Blank absorbance of the ATP and Yeast extract.xlsx
– Data shown in Fig. 3A:
  028 - Change of the absorbance with Alkali-lignin.xlsx
– Data shown in Fig. 3A:
  029 - Change of the absorbance with Ferulic Acid.xlsx
– Blank data not shown in Fig. 2:
  030 - Change of the absorbance with Vanillin.xlsx.

## 2 Experimental design, materials, and methods

The standard mean deviation of the distance of the measured experimental data and the model data, the correlation coefficient *R*^2^ and the analysis of variance (ANOVA) were used for the Evaluation of the model fittings. The single-factor ANOVA were based on a 95% confidence interval for the hypothesis that the experimental and model-derived data are equal. The estimation of the parameter values by model adjustment was performed by the genetic algorithm (GA) using MATLAB (Mathworks, Natick, MA) optimization tools to determine the minimum nonlinear least squares between the experimental and model data.

### 2.1 Optical-density screening

The high-throughput optical-density screening was implemented as described by Glaser and Venus (2014) [2]. Modifications were as follows. A centrifuged inoculum (5,000 rpm, Sigma 4K15 centrifuge, 15 min, 4 °C) was resuspended in a medium containing the variations of glucose, xylose, and glucose with xylose [60 g/L d-(+)-glucose, 60 g/L d-(+)-xylose, 40 g/L d-(+)-glucose with 20 g/L d-(+)-xylose] with 5 g/L yeast extract and 0.025 mol/L sodium acetate buffer pH 6. A set of five alkali-lignin solutions in concentrations of: 0.0, 0.2, 0.4, 0.6, and 0.8 g/L was tested (Lignin, alkali, Sigma-Aldrich Chemie GmbH Munich, Germany). A Bioscreen C from Oy Growth Curves Ab Ltd. was used for the optical-density experiments. Measurements were taken with a wide-band filter (420–580 nm). The Honeycomb plates were prepared as follows. The first three columns were used as medium blanks. In each column, two wells had the same lignin concentrations. Column 4 was used as water blank. Columns 5 to 10 were used to determine the growth. In each case, two columns were used for one saccharide combination and two rows were used for one lignin concentration (Fig. 4). The growth temperature was set to 52 °C. Intermittent-frequency shaking was used with changing amplitudes.

**Fig. 5:**
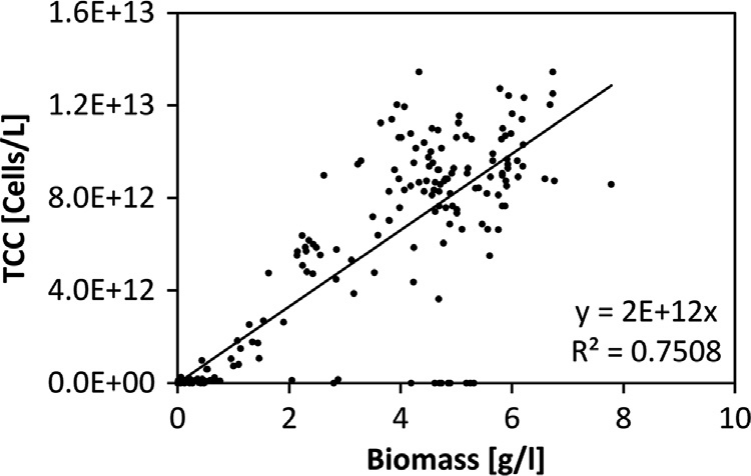
The regression curve of the total cell concentration vs. biomass.

Using the screening tests, a regression curve was developed that defines the relation between the total cell counts (TCC) and the biomass (BM; Fig. 5). The regression is defined by the formula: TCC=1.66E12*BM.

### 2.2 Decolorization by lignin, ferulic acid, and vanillin uptake

The decolorization tests of ferulic acid and vanillin are based on the assays of the ferulic acid decarboxylase activity and vanillin dehydrogenase activity as described in [5], [6], [7]. The modifications were as follows: The three *B. coagulans* strains were cultivated in 5 ml of the MRS medium in culture tubes (52 °C, 15 h). The tubes were centrifuged for 5 min at 5000 rpm (Sigma 4K15 centrifuge). The supernatant was discarded and the cell pellet was washed with sodium potassium phosphate PBS-buffer (pH 7). 0.25 g/L alkali-lignin, ferulic acid or vanillin were solvated in buffer with 0.02 g/L ATP and 0.01 g/L yeast extract. Next, 5 mL of the medium were used to resuspend the centrifuged cells by short vortexing. The tubes with the medium buffer and cells were incubated at 52 °C. Samples were taken at 2.5 h and 5 h. The samples were centrifuged, and the uptake of alkali-lignin, ferulic acid, and vanillin were determined within the UV range of 250 to 400 nm.

## Figures and Tables

**Table 1:** Parameters of growth derived from empirical data (see Fig. 2, Fig. 3, Fig. 4 in Ref. [1]).

KL	DSM No. 2314		DSM ID 14–300			DSM ID 14–301				
	0.0	0.625	0.0	0.625	1.25	0.0	0.625	1.25	2.5	(g/L)
Concentrations of biomass and sugar^a^										
*C*_*BM,min*_	0.03	0.04	0.02	0.04	0.07	0.03	0.08	0.07	0.06	(g/L)
*C*_*BM,max*_	6.09	7.78	4.33	5.92	5.29	5.00	4.81	5.17	5.31	(g/L)
*C*_*Glc,min*_	0.00	0.00	0.00	0.00	0.00	11.90	7.38	0.00	0.00	(g/L)
*C*_*Glc,max*_	46.21	44.64	46.69	45.57	44.58	49.78	47.39	42.76	41.82	(g/L)
*C*_*Xyl,min*_	0.00	0.00	0.00	0.00	0.00	11.27	7.09	11.55	11.16	(g/L)
*C*_*Xyl,max*_	21.81	22.36	21.33	20.68	22.75	21.22	21.84	22.02	22.43	(g/L)
*C*_*Ara,min*_	0.00	0.00	0.00	0.00	0.00	8.81	7.63	8.77	7.07	(g/L)
*C*_*Ara,max*_	10.44	10.76	10.58	10.66	10.95	11.70	10.77	10.58	10.55	(g/L)
*C*_*LA,max*_	68.85	63.71	69.68	67.07	66.00	46.95	50.64	45.56	43.75	(g/L)
*P*^b^	2.648	0.885	2.488	1.397	1.375	0.978	1.055	0.949	0.912	(g/L/h)
*FT*	26	72	28	48	48	48	48	48	48	(h)
*Y*^*BM/Sub*^	0.0776	0.1000	0.0612	0.0682	0.0686	0.0708	0.0555	0.0764	0.0676	(g/g)
*Y*^*LA/Sub*^	0.8775	0.8192	0.5746	0.6329	0.6045	0.5837	0.8591	0.8993	0.8432	(g/g)
*Y*^*LA/BM*^	11.305	8.189	9.391	9.275	8.813	8.239	11.771	15.490	12.475	(g/g)
L-(+)-lactate purity										
*L(+)-LA*	98.89	98.89	99.51	99.70	99.64	99.63	98.93	98.89	98.94	(%)

a Data derived by HPLC measurement described in section 2.3 after inoculum addition. E: Exponent 10.

b The productivity *P* was evaluated as quotient *P*=(*C*_*LA,max*_/*FT*).

**Table 2:** Experimental data and statistical evaluations determining the prediction quality of the model equation under study.

	DSM ID 14 300		DSM No. 2314								
	AM		WSH			AWH		PWH			
	(−)Ara	(+)Ara									
	Fig.2A	Fig.2B	Fig. 2C	Fig.2D	Fig.2E	Fig.2F	Fig.2G	Fig.2H	Fig.2I	Fig.2J	
Concentrations of biomass and sugar^a^											
*C*_*BM,min*_	0.06	0.04	0.25	0.24	0.11	0.43	0.47	0.13	0.14	0.12	(g/L)
*C*_*BM,max*_	2.85	7.22	1.37	1.66	1.52	1.09	1.29	0.39	0.46	0.90	(g/L)
*C*_*Glc,min*_	0.00	0.00	0.00	0.00	0.00	0.00	0.00	0.00	0.00	0.00	(g/L)
*C*_*Glc,max*_	49.37	48.33	7.30	6.80	6.65	5.96	5.56	4.69	4.46	4.90	(g/L)
*C*_*Xyl,min*_	0.00	0.00	0.13	0.00	0.00	0.00	0.00	0.51	0.47	0.00	(g/L)
*C*_*Xyl,max*_	19.36	20.22	2.83	2.21	2.36	1.02	0.97	1.11	0.97	1.54	(g/L)
*C*_*Ara,min*_	0.00	0.00	0.00	0.00	0.00	0.00	0.00	0.00	0.00	0.00	(g/L)
*C*_*Ara,max*_	0.00	10.98	0.63	0.45	0.00	0.00	0.00	0.00	0.00	0.00	(g/L)
*C*_*LA,max*_	57.52	68.81	10.38	9.68	8.88	5.44	5.77	6.10	6.09	6.94	(g/L)

Estimation quality											
*σ*	9.3701	9.4433	1.2630	1.0087	1.2223	1.0824	2.0104	0.8991	1.3413	2.3215	
*R*^*2*^	0.9493	0.9675	0.9741	0.9828	0.9683	0.9460	0.9031	0.9403	0.8913	0.7939	
ANOVA											
*F*	0.0292	0.0267	0.0288	0.0132	0.0006	0.2963	0.1236	0.0313	0.0004	0.0223	
*F*_*critical*_	3.9214	3.9151	4.0426	4.0426	4.0426	4.0195	4.0195	3.9777	3.9777	4.0195	
*p*	0.8644	0.8702	0.8657	0.9090	0.9795	0.5884	0.7264	0.8599	0.9845	0.8818	
